# Distribution of amyloid deposits in the kidneys of a patient with reactive amyloidosis associated with rheumatoid arthritis

**DOI:** 10.1186/1756-0500-6-231

**Published:** 2013-06-14

**Authors:** Takeshi Kuroda, Naohito Tanabe, Hiroe Sato, Takeshi Nakatsue, Yoko Wada, Shuichi Murakami, Masaaki Nakano, Ichiei Narita

**Affiliations:** 1 Division of Clinical Nephrology and Rheumatology, Niigata University Graduate School of Medical and Dental Sciences, 1-757 Asahimachi-Dori, Chuo-ku, Niigata City 951-8510, Japan; 2 Department of Health and Nutrition, Faculty of Human Life Studies, University of Niigata Prefecture, 471 Ebigase, Higashi-ku, Niigata 950-8680, Japan; 3 Department of Medical Technology, School of Health Sciences, Faculty of Medicine, Niigata University, 2-746 Asahimachi-Dori, Chuo-ku, Niigata City 951-8518, Japan

**Keywords:** AA amyloidosis, Amyloid deposition, Distribution, Kidneys, Rheumatoid arthritis

## Abstract

**Background:**

We previously reported that the amount of amyloid A (AA) amyloid deposited in renal biopsy specimens was highly correlated with parameters of renal function. However, the distribution of amyloid deposits throughout the kidneys of these patients, and the degree of renal abnormality, remained unclear. Therefore, we describe the features of reactive amyloidosis associated with rheumatoid arthritis (RA) in an autopsied patient.

**Case presentation:**

The present report case is a 50-year-old female with RA and reactive amyloidosis. She was diagnosed as RA in 1978. Diagnosis of AA amylodosis was made by renal biopsy in 1991 for the reason of proteinuria. Because of the pancreatitis, she was died in 2006 and autopsy was performed. Renal tissues from autopsy specimens were evaluated for their proportions of amyloid-positive areas. A total of 6 specimens (three tissue blocks from each kidney obtained at autopsy) were evaluated in this study. The size of each block was approximately 20 mm × 20 mm. One section of whole tissue was photographed in each case. The borders of the amyloid-positive areas in each specimen were traced in each photograph, excluding any tissue-free spaces. The total amyloid-positive area was measured, and the percentage area of amyloid per whole-tissue section (percent (%) area of amyloid deposition) was calculated. The distribution of amyloid deposits in the kidneys was examined. The significance of differences in the mean percent (%) area of amyloid deposition between the right and left sides and among three long-axis levels (upper, middle and lower) were analyzed by two-way analysis of variance (ANOVA) at a significance level of p <0.05.

**Conclusion:**

The area of amyloid deposition in these samples was about 7-11%, and the degree of variability among them seemed to be small. It also shows a comparison of amyloid deposition between the right and left sides and between the long axis samples for quadruplicate determinations; no significant differences were evident, and thus the percent (%) area of amyloid deposition throughout the whole kidneys appeared to be uniform in this patient.

## Background

Reactive amyloid A (AA) amyloidosis is a serious and life-threatening systemic complication of rheumatoid arthritis (RA) that arises from chronic, systemic and long-lasting inflammation, with elevated levels of serum AA (SAA) protein [1-3]. SAA is an acute-phase 12.5-kDa apolipoprotein associated with high-density lipoprotein, and is the circulating precursor of amyloid A protein. Amyloid A fibrils are insoluble and can be deposited in systemic organs, including the kidneys, heart, or gastrointestinal (GI) tract, owing to the overproduction of SAA under such inflammatory conditions [2-4]. The prevalence of reactive AA amyloidosis in patients with RA is still unclear, but is no longer considered rare. The frequency of AA amyloidosis associated with RA ranges from 7% to 26% [5-9], although the prevalence of clinically symptomatic amyloidosis is reportedly lower [10,11]. Common clinical signs of reactive AA amyloidosis in patients with RA can be found by careful observation for the onset of proteinuria, kidney insufficiency, or GI tract symptoms. We have previously published several reports describing the diagnosis, management and treatment of reactive AA amyloidosis [12-14]. We found that the amount of AA amyloid deposited in renal biopsy specimens was highly correlated with parameters of renal function, such as serum creatinine (Cr) or estimated glomerular filtration rate (eGFR). However, the distribution of amyloid deposits throughout the kidneys of these patients, and the degree of renal abnormality, remained unclear. Here, therefore, we describe the features of reactive amyloidosis associated with RA in an autopsied patient. Our study protocol was approved by the Institutional Review Board of Niigata University Hospital.

## Case presentation

**Figure 1:**
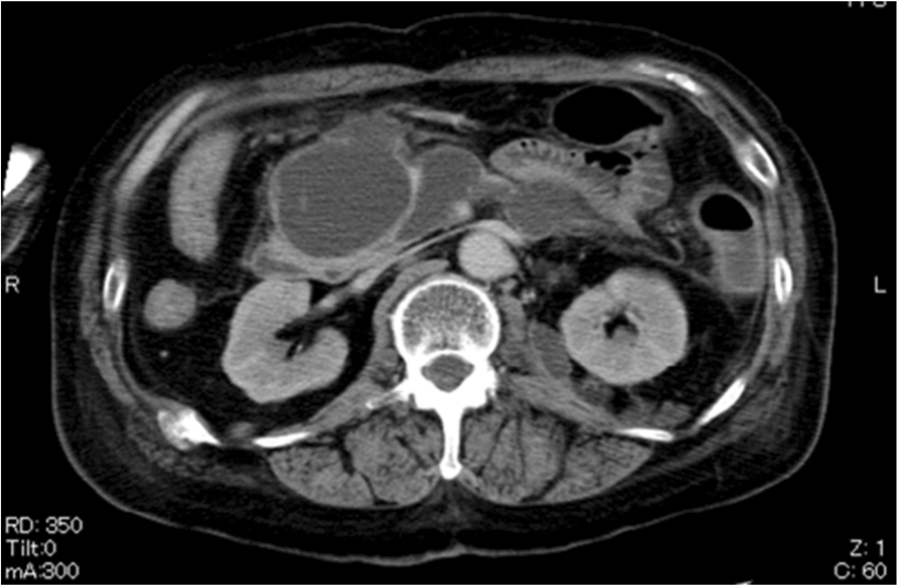
Abdominal CT shows swelling of the pancreas head, multiple pseudopancreatic cysts, and also atrophic kidneys.

A 50-year-old woman was admitted to our hospital for treatment of pancreatitis from our satellite hospital in January 2006. There was no history of either consanguinity or collagen diseases in her family. She had been diagnosed as having RA at the age of 22 years, and had undergone bilateral total hip and bilateral total knee arthroplasty at the ages of 28 and 32 years, respectively. In 1991 she had undergone renal biopsy because of proteinuria, and had been diagnosed as having reactive amyloidosis on the basis of renal biopsy. Her renal function had gradually deteriorated thereafter. Constipation and epigastric discomfort appeared in December 2005, and she was admitted to our satellite hospital because of general fatigue. Laboratory studies revealed a C-reactive protein (CRP) level of 21.9 mg/dL and the amylase (Amy) level (1006 IU/L). Abdominal computed tomogaraphy (CT) revealed swelling of the pancreas head and a pseudopancreatic cyst, and a diagnosis of acute pancreatitis was made. Abdominal CT also revealed that the kidneys were atrophic with a thin renal cortex, but any laterality of the enhancement effect in the cortex was unclear (Figure 1). Her general condition, particularly renal function, deteriorated in spite of intensive treatment. She was transferred to our hospital in January 2006. Laboratory studies revealed CRP 11.0 mg/dL and the blood urea nitrogen concentration was 42.0 mg/dL, and the Cr level was 2.2 mg/dL. Urinalysis showed a protein excretion level of 1.0 g/day, but no hematuria. The rheumatoid factor level was 50.2 IU/mL. Creatinine clearance (Ccr) was 12.7 mL/min per 1.73 m^2^. The levels of trypsin (2112 ng/mL), lipase (130 IU/mL), and DUPAN-2 (440 U/mL) were all elevated. Abdominal CT revealed swelling of the pancreas head and multiple pseudopancreatic cysts and atrophic kidneys. She was started on regular hemodialysis (HD) from mid February. Gradually, HD became difficult to perform because of hypotension, and therefore we switched the patient to continuous hemodiafiltration (CHDF) in March. However, hypotension persisted and she died in early April.

**Figure 2:**
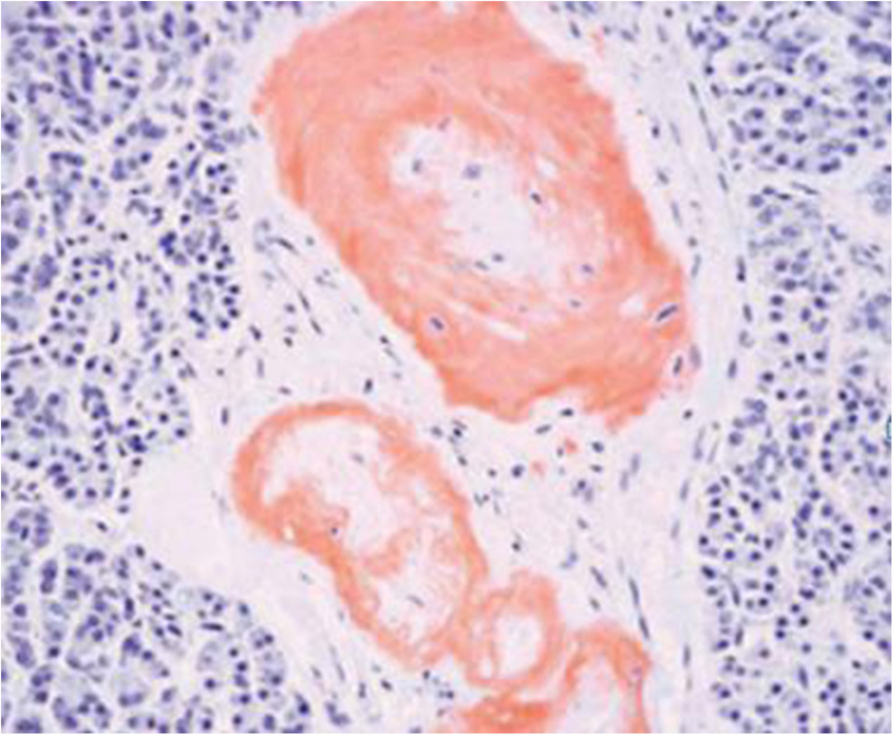
There are many amyloid deposits in the small vessels of the pancreas (Congo red stain).

**Figure 3:**
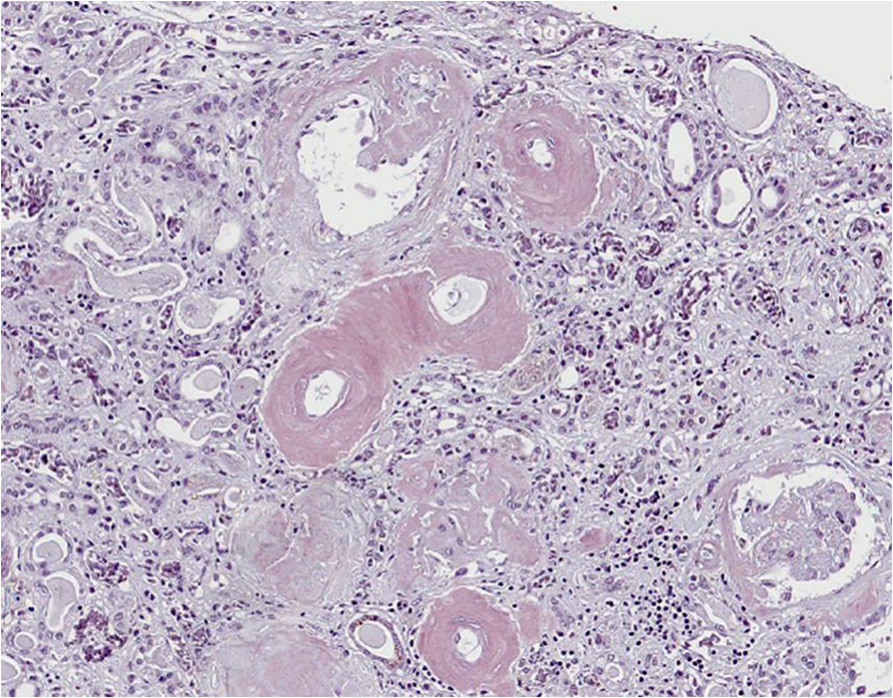
In the kidney, there are many amyloid deposits in the small vessels, whose lumina are narrowed (Congo red stain).

**Figure 4:**
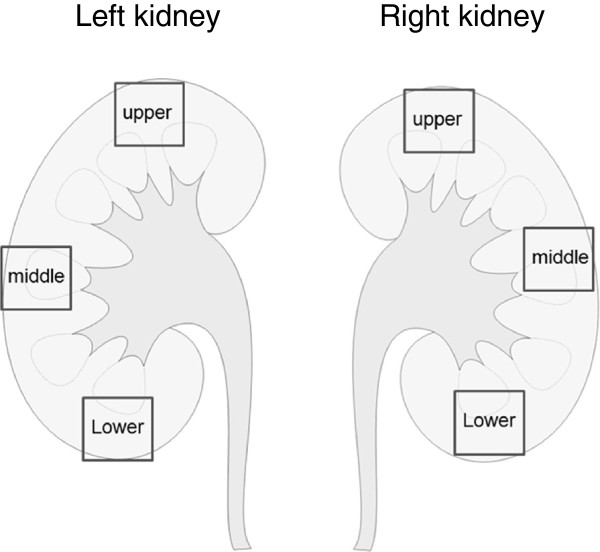
**Schema of specimens taken from the kidney.** A total of 6 specimens (three tissue blocks from each autopsied kidney) were evaluated in this study.

Macroscopic observation at autopsy revealed slightly atrophic bilateral kidneys and a thin bilateral renal cortex. However, the bilateral renal arteries were not obstructed by amyloid deposits. Microscopy revealed reactive AA amyloid deposits in all organs, but mainly in the small arteries. Small-arterial occlusion in the pancreas due to amyloid deposition was evident (Figure 2), and amyloid deposits are noted in interlobular arteries, arterioles, globally sclerotic and sclerosing glomeruli, and thickened Bowman’s capsule (Figure 3). Renal tissues from autopsy specimens were evaluated for their proportions of amyloid-positive areas. A total of 6 specimens (three tissue blocks from each kidney obtained at autopsy) were evaluated in this study (Figure 4). All the specimens were fixed in 10% formalin, embedded in paraffin, and cut into sections 4 μm thick. Sections were considered suitable for quantitative analysis if they contained the full thickness of the renal cortex and medulla, and were cut perpendicularly to the surface. The size of each block was approximately 20 mm × 20 mm. Amyloid-positive areas in the sections were determined by Congo-red staining. One section of whole tissue was photographed in each case. The borders of the amyloid-positive areas in each specimen were traced in each photograph, excluding any tissue-free spaces. The total amyloid-positive area was measured using ImageJ v. 1.47 software (http://rsb.info.nih.gov/ij), and the percentage area of amyloid per whole-tissue section (%) area of amyloid deposition) was calculated [13]. First, each of the 6 specimens was divided into 4, and a total of 24 photographs were evaluated. Second, a rectangular area of the kidney measuring 1.3 mm × 5.0 mm with its long axis perpendicular to the kidney surface was also evaluated. All selected slides were evaluated for the total area showing positivity for amyloid. The significance of differences in the mean percent (%) area of amyloid deposition between the right and left sides and among three long-axis levels (upper, middle and lower) were analyzed by two-way analysis of variance (ANOVA) at a significance level of p <0.05. Table 1 shows the percent (%) area of amyloid deposited in quadruplicate samples. The area of amyloid deposition in these samples was about 7-11%, and the degree of variability among them seemed to be small. It also shows a comparison of amyloid deposition between the right and left sides and between the long axis samples for quadruplicate determinations; no significant differences were evident, and thus the % area of amyloid deposition throughout the whole kidneys appeared to be uniform in this patient. Table 2 shows the percent (%) area of amyloid deposition in the rectangular samples. The long axis of the rectangle was situated in the renal cortex and the short was the luminal diameter of a 16-gauge needle, which is frequently used for renal biopsy. These samples showed areas of amyloid deposition ranging from about 6% to 10% in the cortex alone, with little apparent inter-sample variability. Table 2 also shows a comparison between the right and left kidneys and between the long-axis rectangular samples. No significant correlation between the long-axis samples was evident. However, the left kidney showed a significantly greater area of amyloid deposition than the right kidney.

**Table 1:** Percentage area of amyloid deposition in quadruplicate samples

**Side**	**Long axis**	**Mean (%)**	**SD**	**No. of samples**
Right	Upper	7.21	1.31	4
	Middle	9.49	6.67	4
	Lower	7.58	0.75	4
	Total	8.09	3.72	12
Left	Upper	11.61	3.08	4
	Middle	8.68	2.28	4
	Lower	10.43	4.51	4
	Total	10.24	3.34	12
Both	Upper	9.41	3.21	8
	Middle	9.08	4.64	8
	Lower	9.01	3.36	8
	Total	9.17	3.63	24

P values for main effects of side and long-axis level by two-way analysis of variance were 0.17 and 0.97, respectively.

**Table 2:** Percentage area of amyloid deposition in rectangular samples

**Side**	**Long axis**	**Mean (%)**	**SD**	**No. of samples**
Right	Upper	7.21	1.31	4
	Middle	6.17	0.83	3
	Lower	7.58	0.75	4
	Total	7.06	1.08	11
Left	Upper	10.25	1.81	3
	Middle	8.68	2.28	4
	Lower	7.03	1.7	2
	Total	8.84	2.17	9
Both	Upper	8.51	2.14	7
	Middle	7.6	2.15	7
	Lower	7.4	1	6
	Total	7.86	1.85	20

P values for main effects of side and long-axis level by two-way analysis of variance were 0.03 and 0.40, respectively.

## Discussion

So far, no reported study has investigated the significance of the relationship between the amyloid deposition evident in renal biopsy specimens from patients with AA amyloidosis and clinical parameters such as serum creatinine, urinary protein excretion, and clinical symptoms [15-17]. Recently, we demonstrated for the first time that there were significant correlations between various clinical parameters and the area of amyloid deposition in renal biopsy specimens from patients with AL amyloidosis and also those with AA amyloidosis associated with RA [12-14,18]. There was a significant correlation between the area of amyloid positivity in renal tissue and renal function parameters, especially the Cr level and eGFR, which might serve as potential indicators of the amount of amyloid present in renal tissue. However, those previous studies employed kidney biopsy specimens, and therefore the observations might have been influenced by the distribution of amyloid deposits in the kidney as a whole. In the present study, therefore, we investigated this issue in a single autopsy case of AA amyloidosis. Almost all previous studies of renal amyloidosis have investigated both the renal cortex and medulla, which are usually represented in renal biopsy specimens. Our first study revealed that amyloid deposits were distributed almost evenly, and that there was no difference in terms of laterality or among of the upper, middle and lower parts of the kidneys, in renal biopsy specimens containing the renal cortex and medulla. In other words, our findings suggested that renal biopsy specimens were sufficient for estimation of amyloid deposits. On the other hand, our second study revealed a significant difference between the left and right kidneys, but no differences among the upper, middle and lower parts of the kidneys, at least for the cortex evaluated perpendicular to the surface. Therefore, renal biopsy specimens did not always appear to accurately reflect the situation in the kidney as a whole. The main reasons for this are that renal biopsy specimens are not always obtained perpendicular to the kidney surface, and that they usually contain parts of both of the cortex and medulla. Although, the differences in the amount of amyloid deposits were difficult to explain, differences in blood flow might be at least partly responsible. In our patient, no obstruction of the renal artery was evident macroscopically, and CT imaging of the renal cortex using contrast medium showed no differences in contrast distribution. One possible explanation is that microcirculation in the renal cortex may include many vessels that contain amyloid deposits. These fine microcirculatory differences may influence the deposition of amyloid. However, as the present study involved only a single autopsy case of AA amyloidosis, a larger-scale study of many samples will be necessary to verify our findings.

## Conclusion

We report a case of reactive AA amyloidosis associated with RA. It also shows a comparison of amyloid deposition between the right and left sides and between the long axis samples for quadruplicate determinations; no significant differences were evident, and thus the % area of amyloid deposition throughout the whole kidneys appeared to be uniform in this patient.

## Consent

Written informed consent was obtained from mother of the patient for publication of this case report and any accompanying images. A copy of the written consent is available for review by the Editor-in-Chief of this journal.

## Competing interests

The authors declare that they have no competing interests.

## Authors’ contributions

TK, HS, TN, YW, SM, and MN treated the patient and provided data about the history and laboratory results. TK, NT and MN performed statistical analysis. TK, MN and IN drafted the manuscript. All authors read and approved the final manuscript.
